# Intra-operative identification of a temporal bone fracture line during cochlear implant surgery: a case report

**DOI:** 10.1186/s12887-023-04053-7

**Published:** 2023-05-05

**Authors:** Gaelle Vofo, Sagit Stern Shavit, Ron Eliashar, Michal Kaufmann

**Affiliations:** Department of Otolaryngology- Head and Neck Surgery, Hadassah Hebrew-University Medical Centre, Faculty of Medicine, Hebrew University of Jerusalem, P. O. Box 12000, 91120 Jerusalem, Israel

**Keywords:** Cochlear implant, Intraoperative fracture line, Ossification, Otic capsule involving fracture, Sensorineural hearing loss, Paediatric temporal bone trauma

## Abstract

**Background:**

Temporal bone fractures are divided into otic capsule sparing and otic capsule involving fractures. In the latter, hearing loss, facial nerve paralysis, cerebrospinal fluid leak and meningitis have been reported to occur. The impact of hearing loss can be devastating, especially when occurring in children, with significant risk to speech development and sound localization. In the event of hearing loss, early rehabilitation is therefore of paramount importance. Identification of an intra-operative fracture line with available images and the outcome of such cases has not been reported.

**Case presentation:**

We present the case of a 31-month-old male with an otic capsule involving temporal bone fracture, who presented with ipsilateral profound hearing loss. After all required work-up had been performed, he was admitted for a cochlear implant insertion. Per- operatively, a clear fracture line was seen at the round window niche, but a normal insertion was performed despite the anticipated potential ossification at the fracture line. The dreaded complications of cerebrospinal fluid otorrhea or non-auditory stimulation post-implant did not occur. The peculiarity of this case was its rarity, which was demonstrated by clear images that showed the fracture line on preoperative imaging and intraoperatively.

**Conclusion:**

Cochlear implantation in the presence of a visible fracture line is feasible and the surgical procedure must not be aborted at its discovery. In these cases, post-operative bacterial meningitis can occur and should be treated aggressively with systemic antibiotics to avoid contralateral ossification of the labyrinth due to labyrinthitis.

## Background

Traumatic injuries to the temporal bone with resultant fractures are classified as otic capsule disrupting fractures or otic capsule sparing fractures [1]. In children, road traffic accidents and falls are the most common causes, each accounting for 30 to 50% of temporal bone fractures (TBFs) [2]. Males are three to four times more predisposed to trauma than females [3]. Involvement of the otic capsule is seven times more likely to cause profound hearing loss, four times more likely to lead to cerebrospinal fluid (CSF) leak, and two times more likely to cause facial nerve paralysis when compared to otic capsule sparing temporal bone fractures. This type of fracture may also predispose to intracranial complications in the likes of epidural hematoma and subarachnoid haemorrhage [1].

Sensorineural hearing loss (SNHL) may occur in all cases of head trauma but much more likely in otic capsule involving fractures [1, 4]. Cochlear implants are reported as effective for hearing rehabilitation after TBFs especially in patients with bilateral profound SNHL [5]. In children still undergoing speech and language development, the importance of binaural hearing cannot be overemphasized. The challenges of cochlear implantation after TBFs are the reported occurrence of cochlear ossification, fibrosis, and CSF leak [6]. To remedy these, various techniques of electrode insertion have been reported including various types of cochleostomies, drilling out the obliterated portion of the cochlea, Scala vestibule insertion, and using double array electrodes [7].

However, pre-operative imaging is of paramount importance to anticipate any potential challenges and to eliminate candidates with extensive ossifications and fibrosis [1, 3]. While the fracture-lines are commonly found on computed tomography scans, intra-operative visualization of otic capsule fracture lines are rare and scarcely described. We present the case of a child with an otic capsule involving TBF. During surgical insertion of a cochlear implant, the fracture line was clearly visible but did not impede electrode insertion or implant function after switch-on.

## Case presentation

We present the case of a 31-month-old male. His past medical history including pre- and post-natal periods were uneventful. Family history was positive for genetically inherited hearing impairment amongst his cousins but negative amongst his siblings and parents. He passed the hearing screening with the Otoacoustic Emissions test after birth and his speech and language development were as expected per age. He received all immunization as per his age, including the Anti-pneumococcal vaccines (PREVNAR) in accordance with the national health administration recommendations.

At 13-month-old he suffered from a transverse right temporal bone fracture with otic capsule disruption after a fall from his bed, about one meter high (≈3.3 feet). His immediate post-fall state was complicated by CSF leak that resolved spontaneously. His parents noticed hearing impairment without a change in speech and language development.

His physical examination was unremarkable, without facial nerve weakness, gait disturbances or abnormal findings in otoscopy.

**Fig. 1:**
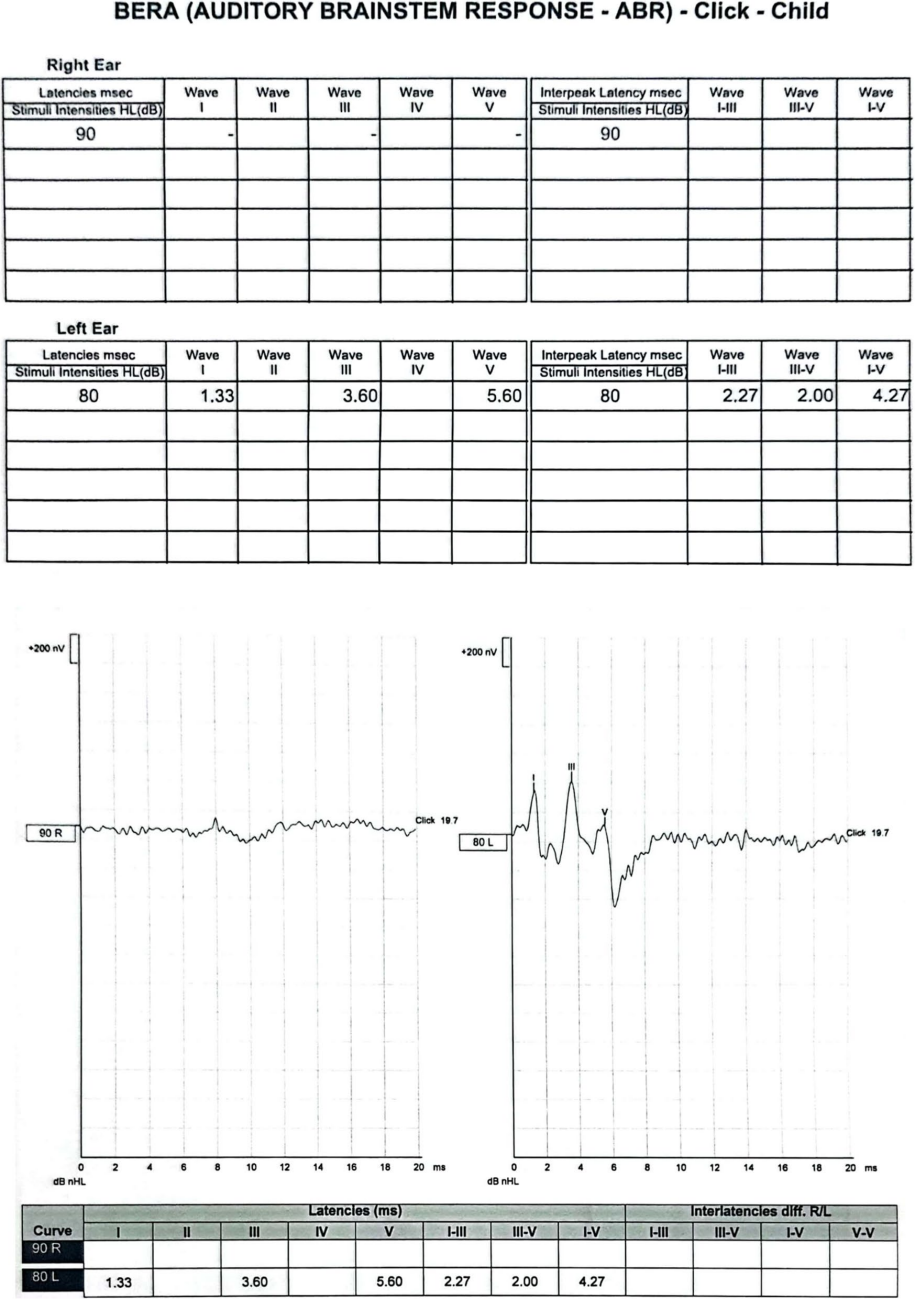
Auditory Brainstem Response test to click stimulus before implantation. Auditory Brainstem Response test demonstrating absent response to click stimulus on the right side and normal response on the left

**Fig. 2:**
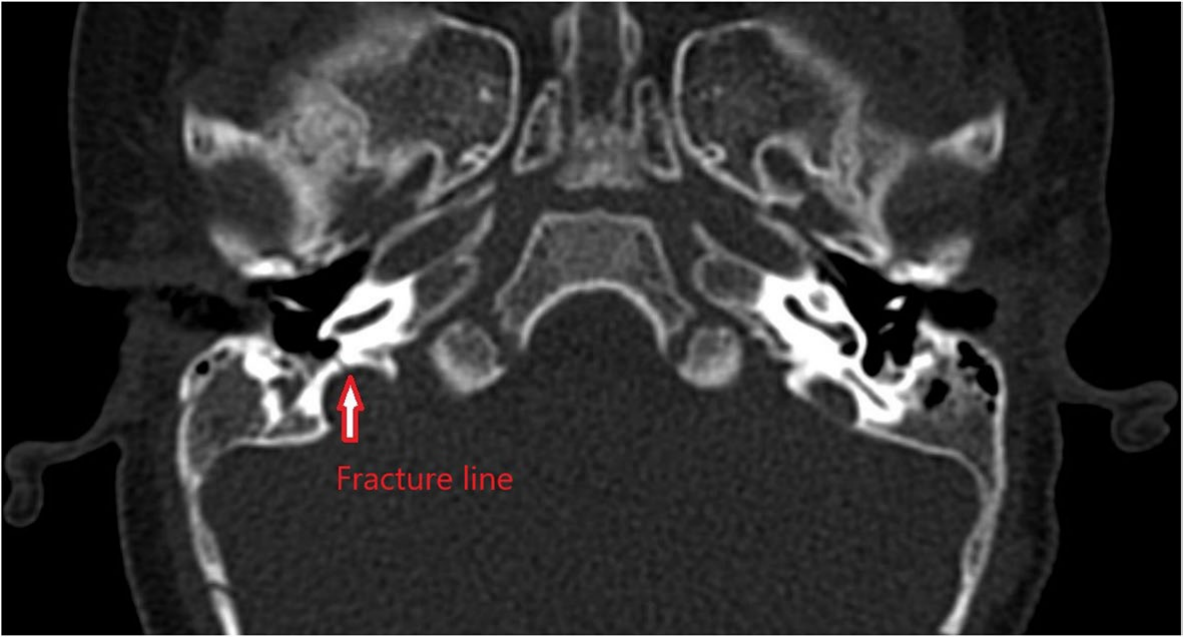
Axial slide of a Computed Tomography scan of the temporal bone without contrast. Axial slide of a Computed Tomography scan of the temporal bone without contrast demonstrating a right transverse temporal bone fracture (red arrow)

**Fig. 3:**
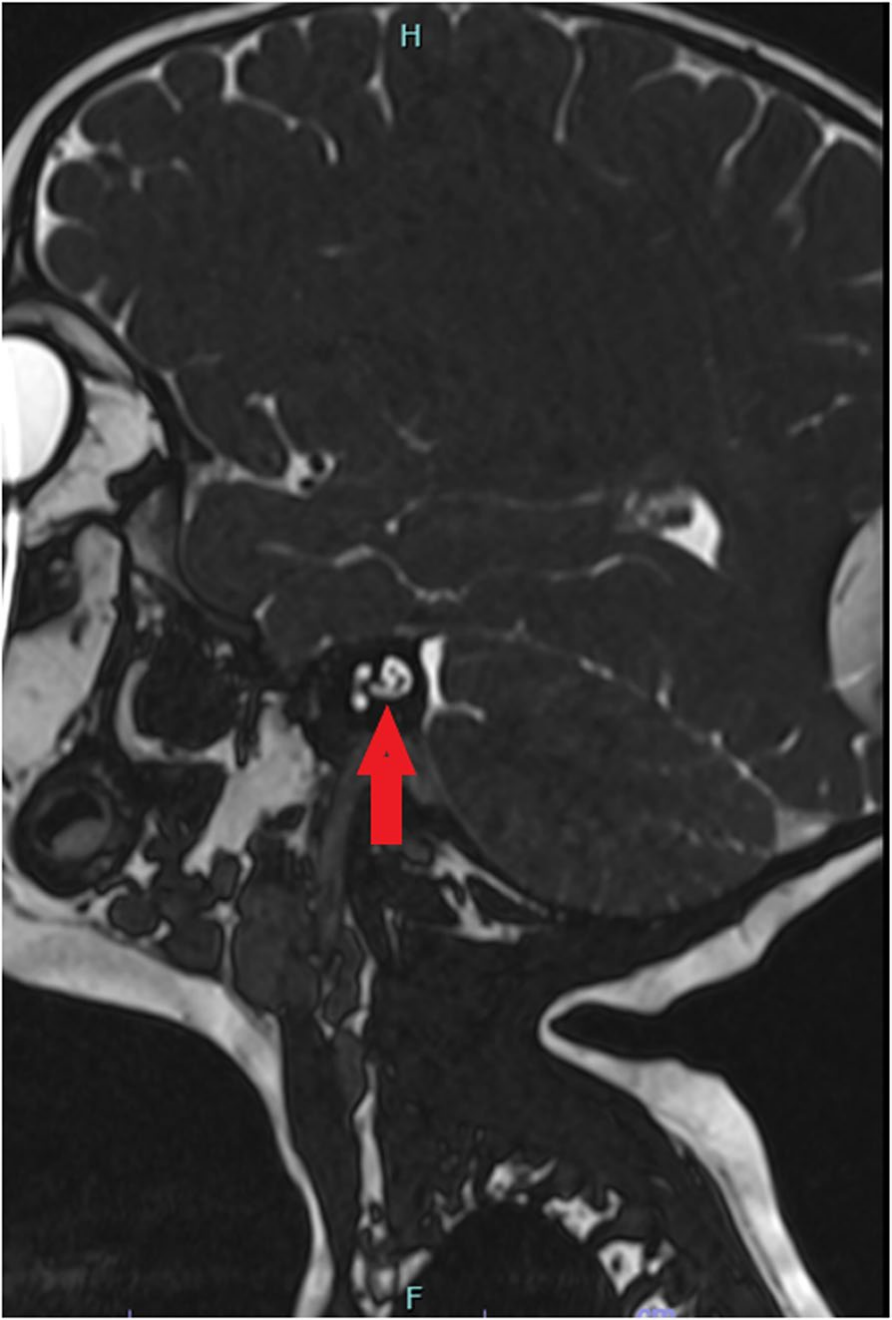
Sagittal T2 Magnetic Resonance Imaging of the head post- trauma. Sagittal T2 Magnetic Resonance Imaging of the head post-trauma with no signs of brain pathology, and the anatomy of the right 7^th^ and 8^th^ cranial nerves was normal (red arrow)

The Auditory Brainstem Response test (Fig. 1) demonstrated absent waves I to V without cochlear microphonics on the right side and normal response on the left. Tympanogram was Type A bilaterally. A computed tomography (CT) scan performed two months post-fall showed a clear transverse fracture in the right otic capsule (Fig. 2) with no signs of cochlea ossification. A Magnetic Resonance Imaging (MRI) (Fig. 3) ten months after the fall (in July 2021) showed no signs of brain pathology, the cochlea lumen and the anatomy of the 7th and 8th cranial nerves were normal. After consideration of all his tests, he was recommended for rehabilitation with a cochlear implant which he underwent in November 2021, that is, thirteen months after the fall.

## Results

**Fig. 4:**
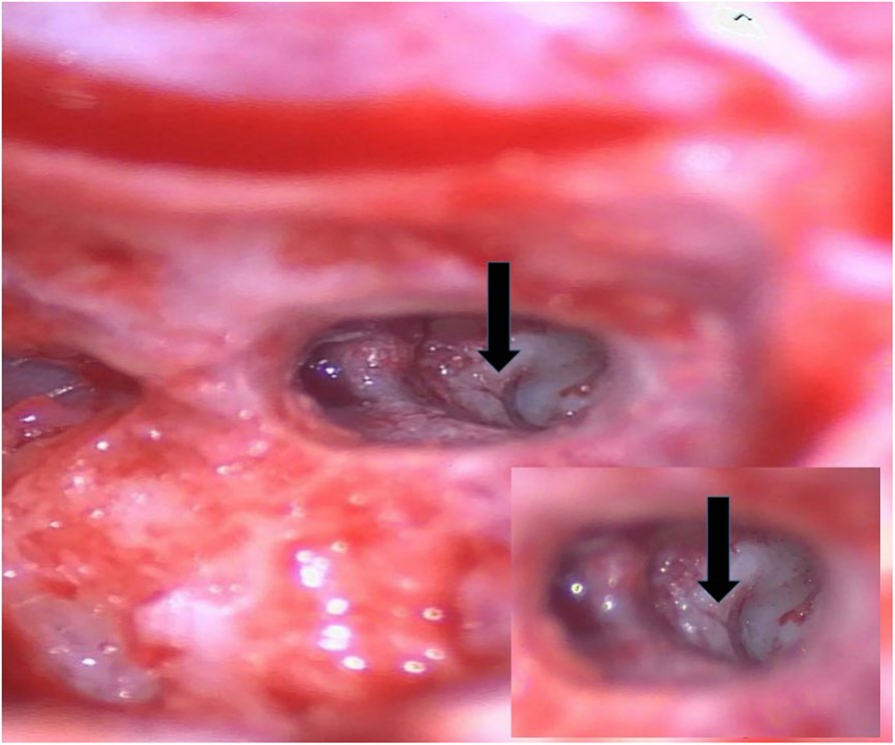
Intra-operative image from a microscope (distant view and closer view). Intra-operative image from a microscope (distant view and closer view) showing the transverse temporal bone fracture line (black arrow)

During the surgery, the fracture line in the round window niche was clearly identified as seen in Fig. 4. A CI622 (Cochlear Nucleus, NSW 2109, Australia) straight slim electrode was inserted via a round window approach without difficulties. No CSF leak was encountered.

**Fig. 5:**
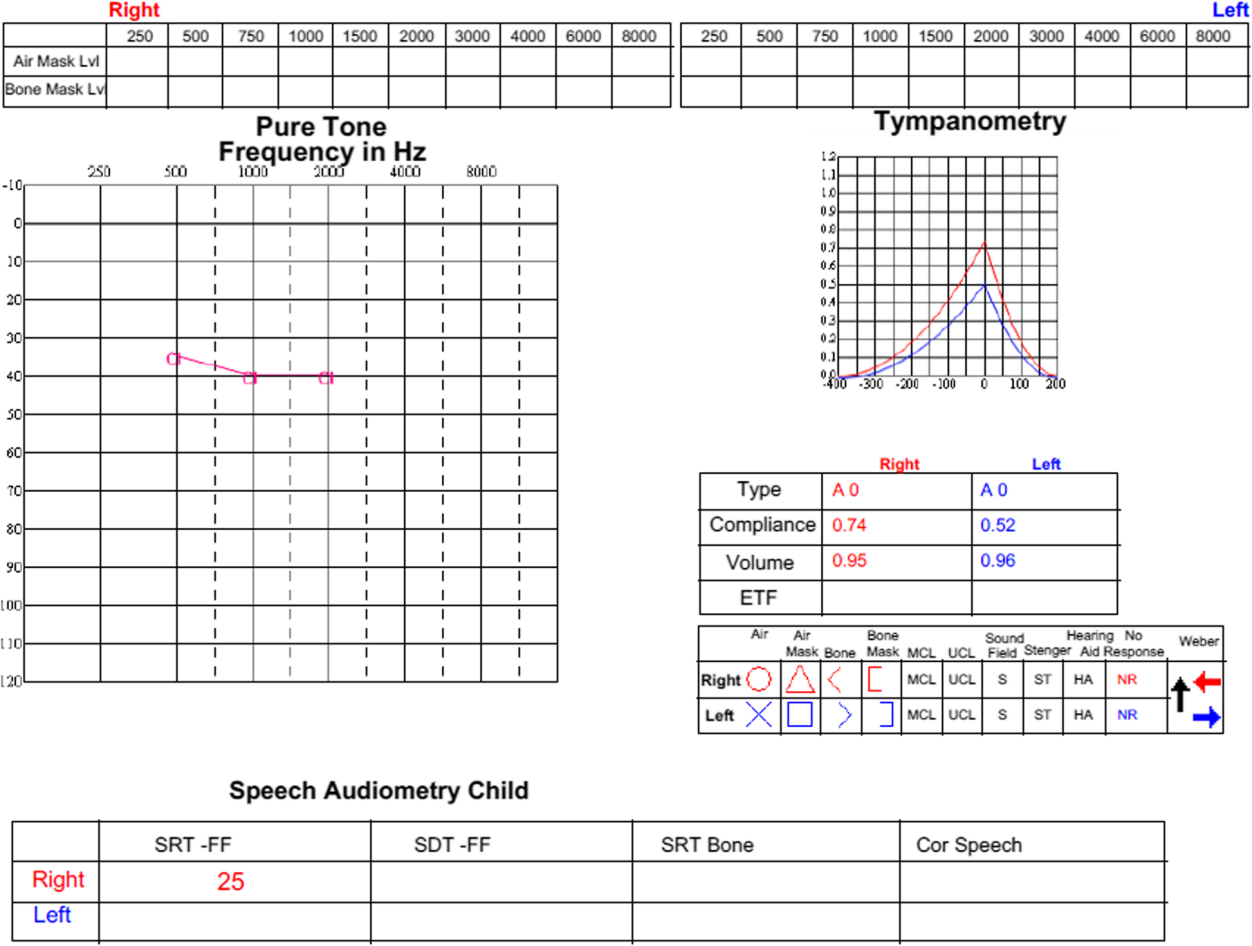
Behavioural Audiometry test with right cochlear implant one year after surgery. Behavioural Audiometry test with right cochlear implant one year after surgery showing improved hearing, with speech reception threshold at free field (SRT-FF) of 25dB and tympanogram type A bilaterally

Twenty days post-surgery he was admitted due to meningitis caused by *Haemophilus influenza* and treated for 10 days with intravenous third generation cephalosporin (Ceftriaxone), followed by prolonged preventive treatment with Amoxicillin for 3 months. Contralateral hearing was not affected by the bacterial meningitis. Switch-on of the cochlear implant was successfully performed in January 2022 and he is currently undergoing calibration sessions with good responses as shown in Fig. 5 by a behavioural audiometry test with the cochlear implant.

## Discussion and conclusions

Otic capsule involving temporal bone fractures are real and their high impact on hearing has been reported. We report the case of a 31-month-old male, whom after a fall incurred a right transverse temporal bone fracture with subsequent ipsilateral profound hearing loss. Of note, this child had not yet fully developed his speech and language.

Cochlear implants have been demonstrated to be effective at restoring hearing in cases of bilateral profound SNHL after traumatic injury, although traumatic and anatomical limitations may make some patients unsuitable for cochlear implantation [5, 6]. An intact auditory nerve which may be injured by traction or avulsion in these injuries must be present for successful implantation [5]. In the paediatric population, more emphasis is laid on the rapid restoration of binaural hearing for proper motor and social developments, as well as speech and language development.

Cochlear implant insertion may be impeded by visible fracture lines, ossification, and fibrosis which all distort anatomy; and also, by a CSF leak [6]. This is more relevant to our case as the cochlear implant surgery was performed thirteen months post-fall in an actively growing child, but neither ossification nor fibrosis were encountered. The absence of ossification was probably because of the high density peculiar to the otic capsule, which may increase the time needed for bone healing, coupled with a stable nondisplaced fracture line with minimal inflammation. The incidence of ossification and/or fibrosis with respect to the duration of an otic capsule fracture has not been reported. Greenberg et al. reported cochlear implantion in eight ears with temporal bone fractures; while those that were implanted had auditory improvement similar to general cochlear implant population without head injury, the authors caution that additional factors such as brain injury severity, and cognitive/behavioural impairments should guide cochlear implant patient selection.

In the event of ossification during cochlear implant insertion, various techniques of electrode insertion have been reported including various types of cochleostomies, drilling out the obliterated portion of the cochlea, Scala vestibule insertion, and using double array electrodes [7]; despite these techniques, failure to fully insert all electrodes has been reported. In this case, intra-operative insertion of a slim straight electrode cochlear implant was uneventful through a standard round window membrane cochleostomy, as no ossification was found around the non- displaced fracture line.

Camilleri et al. reported that fracture lines close to the cochlear implant electrodes can cause non-auditory stimulation secondary to lower electrical impedance along the fracture lines allowing stimulation of the intra-temporal facial nerve [7]. No abnormal facial nerve activities were noted at switch-on and during calibration. Few cases of intraoperative fractures encountered alongside ossifications have been reported but to date [7], no intra-operative images have been shown.

As far as meningitis is concerned, In vitro studies in rats have proven that severe trauma to the osseous spiral lamina and modiolus increased the risk of pneumococcal meningitis even before cochlear implant insertion and that acute electrode insertion did not alter the risk of subsequent pneumococcal meningitis [8]. In the present case, post- cochlear implant meningitis occurred despite adequate immunization and aseptic intra-operative techniques. No studies were found reporting meningitis after cochlear implants in patients with TBFs so we do not have any standard antibiotic recommendations. However, 5 to 35% of patients with bacterial meningitis are likely to develop permanent sensorineural hearing loss, while 4% will develop profound bilateral hearing loss [9] due to multiple factors that include direct labyrinth involvement, cochlear neuroepithelial damage, and vascular insult; thus, placing the contralateral ear at risk. Fortunately, contralateral hearing was not affected in our case.

We present a rare case of a clearly seen intra-operative fracture line in a child with an otic capsule involving transverse temporal bone fracture. Our preamble fear of ossification at the otic capsule thirteen months post trauma was deferred by successful and smooth insertion of the implant electrode. In paediatric cases, the urgency in the management of single side deafness cannot be neglected.

## Conclusion

We conclude that, cochlear implantation in the presence of a visible fracture line is feasible and the surgical procedure must not be aborted at its discovery. Furthermore, our findings both pre-operatively with imaging studies and intra-operatively, confirm the occurrence of hearing loss in otic capsule disrupting TBFs. In these cases, post-operative meningitis can occur and should be treated aggressively with systemic antibiotics to avoid contralateral ossification of the labyrinth.

## Data Availability

Not applicable.

## Abbreviations

CI: Cochlear Implant
CSF: Cerebrospinal fluid
CT scan: Computed Tomography scan
MRI: Magnetic Resonance Imaging
SNHL: Sensorineural hearing loss
TBFs: Temporal bone fractures
